# GEFormerDTA: drug target affinity prediction based on transformer graph for early fusion

**DOI:** 10.1038/s41598-024-57879-1

**Published:** 2024-03-28

**Authors:** Youzhi Liu, Linlin Xing, Longbo Zhang, Hongzhen Cai, Maozu Guo

**Affiliations:** 1 Department of Computer Science and Technology, Shandong University of Technology, Zibo, 255000 China; 2 Department of Agricultural Engineering and Food Science, Shandong University of Technology, Zibo, 255000 China; 3 Department of Electrical and Information Engineering, Beijing University of Architecture, Beijing, 102616 China

**Keywords:** Computational biology and bioinformatics, Drug discovery

## Abstract

Predicting the interaction affinity between drugs and target proteins is crucial for rapid and accurate drug discovery and repositioning. Therefore, more accurate prediction of DTA has become a key area of research in the field of drug discovery and drug repositioning. However, traditional experimental methods have disadvantages such as long operation cycles, high manpower requirements, and high economic costs, making it difficult to predict specific interactions between drugs and target proteins quickly and accurately. Some methods mainly use the SMILES sequence of drugs and the primary structure of proteins as inputs, ignoring the graph information such as bond encoding, degree centrality encoding, spatial encoding of drug molecule graphs, and the structural information of proteins such as secondary structure and accessible surface area. Moreover, previous methods were based on protein sequences to learn feature representations, neglecting the completeness of information. To address the completeness of drug and protein structure information, we propose a Transformer graph-based early fusion research approach for drug-target affinity prediction (GEFormerDTA). Our method reduces prediction errors caused by insufficient feature learning. Experimental results on Davis and KIBA datasets showed a better prediction of drugtarget affinity than existing affinity prediction methods.

## Introduction

The global pharmaceutical industry today is facing enormous challenges. Intense product competition, patent expiration, shorter exclusivity periods, and price constraints pressure pharmaceutical companies to reduce costs , increase productivity, and accelerate growth^1,2^. It takes companies more than $500 million and approximately 12–15 years to bring new compounds to market^1,3–5^. Less than 5% of all compounds screened enter preclinical development, and only 2% of these candidates enter clinical testing^1,4^. Approximately 80% of all drugs that enter phase I trials fail in development^1^. To address these challenges, many research institutions and pharmaceutical companies have turned their attention to the drug repositioning model^6^, which involves analyzing the economic benefits and drawbacks identified by experts. Therefore, we are strongly motivated to develop a computational model that can predict the affinity of new drug-target pairs based on previously existing drugs and targets.

Drug-target affinity (DTA) prediction is crucial for speeding up the drug screening process. Various computational methods^7^ have been proposed for this purpose. Mainstream methods include ligand/receptor-based methods^8^, gene ontology-based methods^9^, text mining-based methods^10^, and reverse docking methods^11^. These methods are continuously being improved under different conditions. Receptor-based methods often employ docking simulations^6,12^, which require 3D structures of target proteins^13^. However, obtaining such structures can be expensive and challenging. Ligand-based approaches suffer from poor predictions when the number of known ligands for the target protein is small. This approach relies on the similarity between candidate ligands and known ligands. Gene ontology-based and text mining-based approaches face similar limitations due to the content reported in the text. Moreover, redundant names of drugs and target proteins complicate these methods. Text mining methods are also limited to existing academic literature, making it difficult to discover and acquire new knowledge.

Machine learning has addressed limitations over time. For instance, SimBoost models utilize known drug association/similarity networks and known target protein association/similarity networks to create new features for predicting the DTA of unknown drug-target pairs^14^. Alternatively, similarity can be derived from other known information instead of training data affinity. Kernel-based approaches, such as regularized least squares regression (RLS) with kernels constructed from drug and target molecular descriptors, are used^15^. KronRLS models are calculated from the Kronecker product of drug and protein kernels into pairs of K^15^ (any similarity metric can be used) to speed up model training. Predicting drug-target interactions (DTI) can also aid in DTA prediction. Research in this area includes DTI-CDF^16^ (a cascaded deep forest model), MLCLB^17^ (a new multi-label classification framework), and DTI-MLCD^18^ (multi-label learning to support community detection).

Some approaches utilize shallow neural networks on drugs and proteins. DeepAffinity^19^ employs seq2seq self-encoders^20^ for unsupervised learning of protein and compound feature representations. The learned encoder’s output is then passed to the attention layer and further to the 1D convolution layer. The outputs of the protein and compound convolutional layers are combined and fed into the fully connected layer. Similarly, the DeepDTA^21^ model adopts a 1D representation and a 1D convolutional layer (with pooling) to capture data patterns. The final convolutional layers are connected, followed by multiple hidden layers, and regression is performed using drug-target affinity scores.

Deep learning models are among the best-performing models for DTA prediction. Many works^22–25^ have been carried out in deep learning models. However, these models use drug SMILES as direct input, which may not capture the complete uniqueness of the molecular structure of drugs. By using data in string format, molecular structure information is lost, which may reduce the functional relevance between potential drug molecules, which in turn can reduce the predictive power of the model. The development of graph convolutional neural networks^26,27^ has migrated from other fields to biological information. It has been used for drug discovery^28^ , including interaction prediction^29^, affinity prediction, synthesis prediction, and drug repositioning^30^. Since protein biomechanics inherently contains more structural information, previously proposed methods mainly use protein sequence information directly as input to the model, and these methods lose a large amount of protein structural information.

This paper introduces GEFormerDTA, a novel neural network model that integrates drug and protein structure information. It leverages four forms of feature representation (node, degree center, space, and edge encoded features) to effectively utilize their roles in the graph task. Secondary structure information and ASA information of the target protein are incorporated, enabling comprehensive utilization of protein structural information. An early fusion mechanism is employed to handle the binding affinity between drugs and proteins, reducing prediction errors caused by information redundancy.

## Materials and methods

### Problem definition

The drug-target binding affinity (DTA) problem aims to predict the binding affinity between a drug and a target protein. This is a mathematical regression problem:

1 $$\begin{aligned} A={\mathscr {F}}_\theta (P,D), \end{aligned}$$

where $D=\{d_1,d_2,d_3,\ldots ,d_i\}$, $P=\{p_1,p_2,p_3,\ldots ,p_i\}$, and $\theta$ is a learnable parameter in the prediction model ${\mathscr {F}}$. Our task is to predict the affinity score between $t_i$ and D or T and $d_j$, given a new drug $t_i$ and target protein $d_j$.

### Dataset

We evaluated our proposed model on two different datasets, the kinase dataset Davis^31^ and the KIBA dataset^32^, both of which have been used as gold standard datasets for prediction assessment in DTI and DTA studies^14,33^.

**Table 1:** Dataset summary.

	proteins	drugs	links
Davis ($K_d$)	422	68	30056
KIBA	229	2111	118254

**Figure 1:**
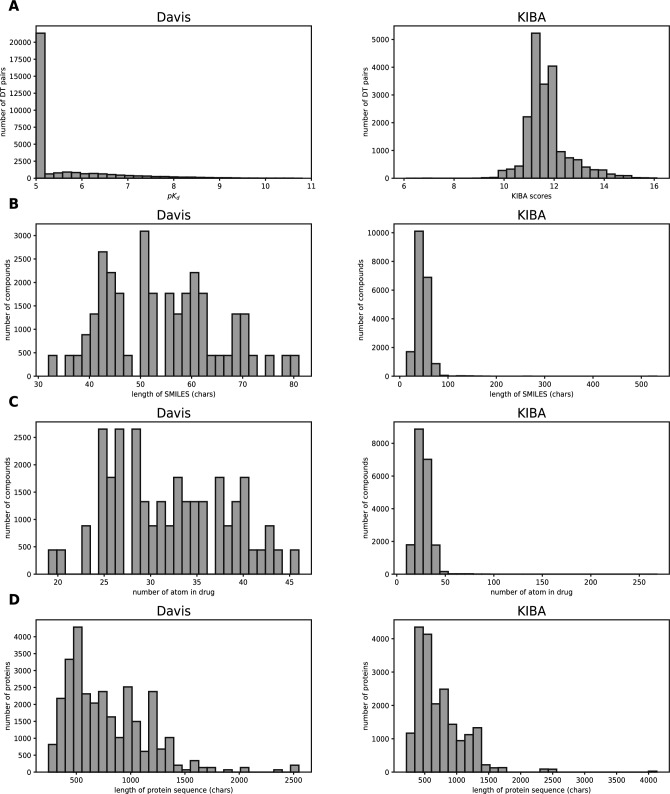
Summary of the Davis (left panel) and KIBA (right panel) datasets. (**A**) Distribution of binding affinity values. (**B**) Length distribution of SMILES strings. (**C**) The number of atoms of drug molecules. (**D**) Length distribution of protein sequences.

The Davis dataset contains selective assays of kinase protein families, related inhibitors, and their respective dissociation constant ($K_d$) values. It contains the interactions of 442 proteins and 68 ligands. On the other hand, the KIBA dataset was derived from a method called KIBA, which combines the biological activities of kinase inhibitors from different sources (e.g., $K_i$, $K_d$, and IC50)^32^. The study of predicting these kinase inhibitors can be explored through^34^. KIBA scores were constructed to optimize the concordance between $K_i$, $K_d$, and IC50 by exploiting the statistical information they contain. The KIBA dataset initially contained 467 targets and 52498 drugs^14^. Removing these drugs and targets can mitigate the impact of noise on model training, balancing the dataset and preventing an undue focus on specific drugs and targets during the model training process. Tables 1 summarizes these datasets we used in our experiments. To demonstrate the properties of the drugs and proteins more visually in Table 1, we depict the breadth and length of the two gold standard data through Fig. 1.

Regarding data density, the model performs well in handling sparse graphs, considering only the immediate neighbors of nodes. Therefore, the model performs better when dealing with the low-density KIBA dataset. However, its performance is poorer in the high-density Davis dataset. Concerning data size, the model utilizes self-attention mechanisms to handle small-scale data, capturing global information about the molecular graph neighborhood and aiding in extracting key node information. However, when dealing with large-scale data, the model has longer training cycles.

While^33^ directly uses the $K_d$ values from the Davis dataset as binding affinity values, we employ the transformed values into logarithmic space, denoted as $pK_d$ , similar to the equation (2) described.

2 $$\begin{aligned} {\text {p}}K_{\text {d}}=-\log 10\left( \frac{K_d}{1e9}\right) , \end{aligned}$$

### Drug representation

In the dataset, the pairs of affinity primarily consist of drugs and proteins. The input for drug compounds mainly utilizes two data formats: SMILES and SDF. In our proposed method, the molecular graph of a drug is constructed based on the SMILES string and SDF file data. Specifically, the SDF format molecular data is parsed using the RDKit tool^35^ to obtain the two-dimensional structural information of the molecule. In the molecular graph representation, atoms represent the nodes of the graph. The combination of node features encompasses a variety of properties, including atom symbol, atom degree, atom implicit valence, the number of free valence electrons, atom hybridization type, and atom aromaticity. These attribute features are concatenated to form a multidimensional feature. The edges in the graph represent the chemical bonds of the molecule, and the presence or absence of an edge between two nodes indicates whether there is an interaction between the atoms. We construct an adjacency matrix based on these edges, which encapsulates the positional information of the node with respect to other nodes. In our study, we use ${{\mathscr {G}}}_{d}=({{\mathscr {V}}}_{d},{{\mathscr {E}}}_{d})$ to represent the graph representation of the drug compound, where ${{\mathscr {V}}}_{d}$ represents the atoms of the drug compound, and ${{\mathscr {E}}}_{d}$ represents the chemical bonds of the drug compound.

We define the set of attributes of atom j of the i-th drug $d_i$ in the entire drug set D of the database as ${x}_{j}^{d_{i}}$, which is a vector of nine attributes, denoted as follows:

3 $$\begin{aligned} {x}_j^{d_i}=[a_1,a_2,\ldots ,a_9], \end{aligned}$$

where ${x}_{j}^{d_{i}}$ represents the mathematical expression of atom j of drug $d_i$, $a_1$ represents the number of atoms in drug $d_i$, $a_2$ represents chiral information including R-type, S-type, axial chirality, planar chirality, and helical chirality, and $[a_3,a_4,\ldots ,a_9]$ represents, in order, the atomic degree (number of chemical bonds), formal charge, number of connected hydrogen atoms, free radical number of electrons, type of atomic hybridization, whether or not an aromatic bond is formed, and whether or not an a-ring is present. ${x}_{j}^{d_{i}}$ in these properties can be obtained by the RDKit tool and embedded as integers under the guidance of a predefined dictionary.

#### Degree centrality encoding

**Figure 2:**
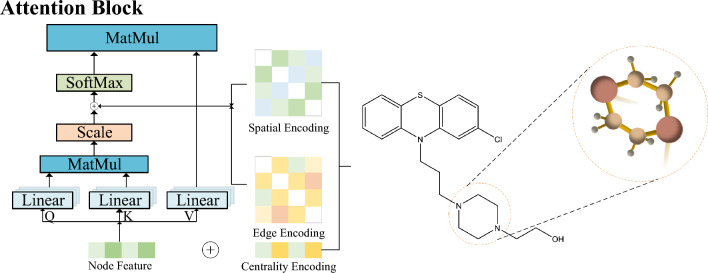
Diagrammatic representation of centrality coding, spatial coding and edge coding used for the structure of drug molecules.

We first extracted the atomic and chemical bonding information of the drug using the RDKit tool^35,36^. The more edges an atom exists, the more critical the atom becomes, or the more complex the interconnections with other atoms are to the model. In this paper, we characterize the degree features in the molecular graph by atomic degree centrality as an additional signal for the neural network. Since the degree centrality habit encoding (see Fig. 2) is used for each node, we only need to combine it with the atomic node corpora to form the degree centrality features of the atoms. This encoding allows the model to capture the semantic relevance and importance of the atoms more confidently and pass them into the attention mechanism, as shown in the following mathematical equation:

4 $$\begin{aligned}{} & {} h_j^{d_i}={x}_{j}^{d_{i}}+e_{\deg ^{-}(v_j)}^{-}+e_{\deg ^{+}(v_j)}^{+},\end{aligned}$$

5 $$\begin{aligned}{} & {} Featru e_{deg_{ij}}=\frac{(h_i W_Q)(h_j W_K)^T}{\sqrt{d}}, \end{aligned}$$

where $e^{-}$, $e^{+}\in {\mathbb {R}}^d$ denote the incoming and outgoing degrees of atomic nodes specifying the learnable embedding vectors, respectively, Additionally, $h_j^{(d_i)}$ denotes the atomic features of atom j in drug $d_i$. Here, *d* denotes the modulation factor, and $W_Q$ and $W_K$ are the weight matrices for atoms (nodes) i and j, respectively.

For undirected graphs, the incoming degree ${deg}^{-}\big (v_j\big )$ and outgoing degree ${deg}^{+}\big (v_j\big )$ can be uniformly denoted as $\deg (v_j)$. By adding the degree-centric encoding feature to the nodes, softmax attention can capture the critical information of the nodes in K and Q. Therefore, the model can capture the semantic relevance and the critical information of the nodes in the attention mechanism.

#### Atomic spatial position encoding

The Transformer possesses globality, but it relies too heavily on positional information for encoding. When solving sequential data present in natural language problems, it is possible to encode each position (i.e., absolute position encoding)^37,38^ or to encode any two positions in the Transformer layer (i.e., relative position encoding)^39^.

However, when we use the graph information built based on the spatial structure as the input to the Transformer model, it is instead detrimental to the prediction of the model. We introduce the spatial location encoding to capture the spatial structure information of the drug graph. First, we write down the set of drug nodes as ${\mathscr {V}}_{d}=\{v_{j}\mid v_{j}\in {\mathbb {R}}^{N}\}_{j=l}$, given a function $\phi (v_i,v_j)\in {\mathbb {R}}^N$ representing the spatial relationship between $v_i$ and $v_j$. We describe the function $\phi (v_i,v_j)$ as a connectivity definition graph between nodes. In the drug diagram, we set the pathway $\phi (v_i,v_j)\in {\mathbb {R}}^N$ between $v_i$ and $v_j$ to denote

6 $$\begin{aligned} \phi \big (v_{i},v_{j}\big )=\left\{ \begin{matrix}SPD(v_{i},v_{j}), &{} | &{} v_i\rightarrow v_j\\ -1, &{} | &{} v_{i}\not \rightarrow v_{j}\end{matrix}\right. , \end{aligned}$$

where SPD($v_i$, $v_j$) denotes the shortest dependency path (SDP) reachable between $v_i$ and $v_j$.

After we encode by degree center and spatial location, we obtain the embedding matrix of the atomic pair (node pair) ($v_i$, $v_j$) as

7 $$\begin{aligned} Featrue_{p_{ij}}=W_{\phi }^{(i,j)}\phi \bigl (v_i,v_j\bigr ), \end{aligned}$$

where $W_{\phi }^{(i,j)}$ is the weight of the spatial location feature of the drug node pair, and $Featrue_{p_{ij}}$ is the embedding of the spatial structure feature.

#### Interatomic chemical bonding coding

Edges are also an important component in handling graph tasks. For example, in molecular graphs of drug compounds, features describing the types of chemical bonds can be assigned to atom pairs. These features are as crucial as node features in representing the graph and are indispensable for encoding in graph tasks. Previous approaches to graph tasks mainly include two methods: (1) Edge features are added to the associated node features^40^. (2) For each node, the features of its associated edges are used together with the aggregated node features^41^. However, these approaches only propagate edge information to their associated (neighbor) nodes, which may not effectively utilize edge information to represent the entire graph.

We introduce atomic compound chemical bond encoding to encode edge features into the attention layer better. For the adjacent atom-pairs edge encoding approach is defined:

8 $$\begin{aligned} e_{\left( v_{i},v_{j}\right) }=[b_{1},b_{2},b_{3}], \end{aligned}$$

where $b_1$ denotes the bond type, $b_2$ denotes the steric bond, and $b_3$ denotes whether the bond is conjugate. $b_1$, $b_2$ and $b_3$ can be obtained by the RDKit tool. If the shortest path of i and j is ${{\textbf {P}}}=(e_1,e_2,\ldots ,e_k)$, then

9 $$\begin{aligned} Featrue_{e_{(v_i,v_j)}}=\frac{1}{k}\sum _{{\text {t}}=1}^{\text {k}} W_{{\text {edge}}}{{\textbf {P}}}_{\text {t}}. \end{aligned}$$

### Protein representation

Previous studies^25,42^ typically used protein sequences as input for deep learning models, where protein residues were encoded into a vector space using techniques like one-hot encoding or BPE encoding. These studies employed a lightweight 1D convolutional layer encoder to extract valuable features from the protein. However, these methods solely captured the primary structure information of proteins. Predicting the 3D structure from a 1D sequence is a formidable task, making 1D representations inadequate for capturing the spatial structural features of proteins. Obtaining 3D structures for certain proteins is challenging due to their limited representation in databases^43^. Moreover, representing the irregular 3D structure requires a large-scale 3D matrix, resulting in computationally expensive model execution. Additionally, experimentally determined 3D structures may suffer from low quality since they depend on the intricate and demanding process of co-crystallization of protein-ligand pairs. Hence, it is necessary to shift our focus towards the secondary structure and other protein information.

To tackle the complexity and accessibility challenges, we employ SS and ASA^44^ for representing the protein graph structure. SS determines the backbone structure of the target protein, while ASA indicates the degree of contact or exposure of amino acid residues to the solvent in its three-dimensional structure. The interaction between non-adjacent residues is denoted as DM, which serves as a protein feature. The pairwise distance matrix of residues efficiently captures contact information in the protein structure and can be calculated using SPOT-Contact^45^.DM has proven successful in predicting various protein spectra, such as solubility^46^, DTI^47^ and DTA. Contact between two non-adjacent residues occurs when their distance is less than 8 ${{\text{\AA }}}$. However, simply vectorizing each residue in the protein sequence using unique thermal encoding lacks information about element similarity and treats them as equal in distance. This representation also limits the model learning capability by disregarding the dependency information between residues. In many protein datasets, only a limited number of target proteins provide available information, while most of the protein information remains untapped, leading to detrimental DTA prediction results.

**Figure 3:**
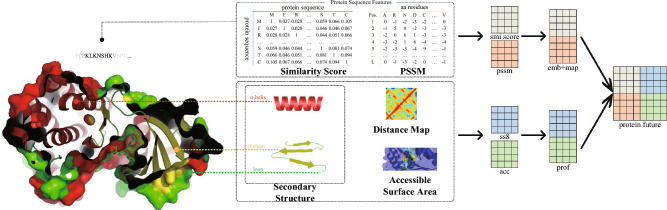
Summary of protein features that can be used to study drug target interaction affinity.

The TAPE^48^ approach utilizes amino acid embeddings in a continuous vector space and employs the self-attention mechanism of the Transformer to capture contextual relationships and information in protein sequences. Instead of one-hot encoding, TAPE uses embedded representations learned from unlabeled protein sequences to represent protein graph nodes. Fusion of embedding vectors from TAPE, secondary structure, and solvent accessibility feature vectors represents node features in the protein graph (see Fig. 3). Each amino acid residue is assigned to one of eight categories, providing detailed secondary structure information. Given a protein sequence of M residues, the node feature set ${\mathscr {V}}_{p}=\{v_{i}\mid v_{i}\in {\mathbb {R}}^{h}\}_{i=l}^{M}$, where h is the length of the embedding vector $v_i$ provided by TAP, captures context-dependent residues. Protein secondary structure, formed by coiled folding of peptide chains, contains vital information about protein activity, function, and stability, benefiting model predictions. Distance map as global structure information may be important in future DTA identification.^47^ introduced super nodes connecting other nodes in the composite structure graph.

### Proposed model

**Figure 4:**
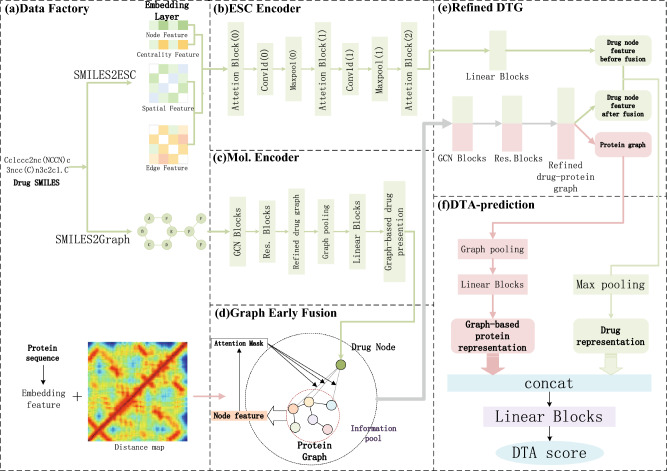
Diagram of the proposed model architecture. (**a**) is the data pre-processing stage of the proposed model. (**b**) is the encoder of the drug ESC. (**c**) is the encoder of the drug graph. (**d**) is our proposed graph feature early fusion process. (**e**) is the drug-target protein graph refinement process. (**f**) is our DTA final prediction process.

The general architecture of our proposed method is shown in Fig. 4. Our GEFormerDTA takes the drug molecule graph structure $G_d$ and the target protein graph structure $G_p$ as inputs and outputs the final prediction results. In processing the graph structure information, we use a graph convolutional neural network model (GCN). Our GEFormerDTA model consists of five main key steps: information preprocessing (Fig. 4a), drug ESC encoding (Fig. 4b), drug Graph encoding (Fig. 4c), drug-target protein graph early fusion (Fig. 4d), drug-target protein graph refinement (Fig. 4e) and affinity scoring (Fig. 4f). In the steps of Fig. 4b,c,e, we also added residual jumps to slow down the generalization performance of our network.

#### GEFormerDTA overview

Before we input the drug into the GEFormerDTA model, we need to encode the drug by two types of encoders: (1) ESC encoder; (2) Mol. encoder. For the ESC encoder, we mainly use the global sensory field of the Transformer to capture the global information of the drug molecule, while the Mol. encoder captures the main node information in the drug graph information. Meanwhile, we fuse the obtained protein feature maps with the drug feature maps extracted by the Mol. encoder features. The fused drug-protein fusion map is fed to the drug target protein fractionation process to obtain the fractionated drug-protein map, and finally the results are obtained by DTA prediction.

#### ESC encoder

As shown in Fig. 2, after obtaining the node features, spatial position features, and edge features of the molecular graph, if we use traditional attention models, we will face the challenge of high dimensionality and many molecular nodes, which seriously affects the efficiency of model training. In addition, to address the issue of memory overhead, we introduce the Sparsepro self-attention molecular graph encoder to extract important Q and reduce model complexity. Meanwhile, we use self-attention distillation to reduce feature dimensionality and the number of network parameters. As shown in Fig. 4b, our drug molecule encoder is a sandwich model that includes 3 layers of Sparsepro self-attention and 2 layers of GCN. Our Sparsepro self-attention can attach great importance to atoms or edge matrices that make significant contributions, while ignoring others. Sparsepro self-attention can be expressed by the following mathematical formula:

10 $$\begin{aligned} {\textbf {At}}{\textbf{n}}(Q,K,V)={\textbf {Soft}}\textbf{max} \bigg (\frac{{\bar{Q}}K^T}{\sqrt{d}}\bigg )V, \end{aligned}$$

where ${\bar{Q}}$ is a sparse matrix of the same size as Q, which contains only top-S queries. We compute all queries in Q and sort them based on the sparsity of KL scattered points^49^. This paper adopts S=25 to form ${\bar{Q}}$ and replace Q. The time complexity of point-wise computation in Sparsepro self-attention is $O\big (ln L_Q\big )$, and the memory usage for each Q-K lookup and each block is $O \left( L_K ln L_Q\right)$^49^. After improving Formula (10), we obtain the following expression:

11 $$\begin{aligned} Featru e_{deg_{ij}}=\frac{(h_i W_{{\bar{Q}}})(h_j W_K)^T}{\sqrt{d}}, \end{aligned}$$

After inputting all the features of the drug molecule graph into the model, we employ an expression to calculate the self-attentiveness of Sparsepro is

12 $$\begin{aligned} \begin{aligned} {\textbf {Attn}}_{(i,j)}&={\textbf {Softmax}}\Big (Featrue_{deg_{ij}} +Featrue_{p_{ij}} + Featrue_{e_{(v_i,v_j)}}\Big )(W_V h_i), \end{aligned} \end{aligned}$$

In addition, we set a GCN distillation operation immediately after each Sparsepro self-attentive block to prioritize mappings with focal features and capture the focal feature map as input at the next layer. The specific operation flow equation is as follows:

13 $$\begin{aligned} X_{j+1}=MaxPool\bigg (ELU\bigg (Conv1d\bigg (\left[ X_j\right] _{ops} \bigg )\bigg )\bigg ), \end{aligned}$$

where $[\cdot ]_{ops}$ denotes the output of Sparsepro self-attentive block after having some column operations, $X_j$ denotes the input of the j-th self-attentive block, Conv1d denotes the 1D convolutional layer, ELU is the activation function, and MaxPool is the maximum pooling layer.

We need to transform the SMILES sequence of the drug into a 2D structure by scripting before inputting the drug into the GEFormerDTA model, and then we extract the atomic structure information from the 2D structure information of the drug, after which we convert the atomic information into an information encoding that can be applied to the attention mechanism by^50^ three encoding designs.

#### Mol. encoder

For the accuracy of model prediction, we also leverage the graph information of drug molecules as inputs to the model. This approach differs from the treatment of drug data mentioned in 2.5.2, where the atomic features of the drugs (element types, atomic degrees, atomic indices, atomic implicit valence, formal charge, hybridization types) are directly fed into the Mol. encoder.

Due to the strong affinity of GCN networks for graph information, we use the GCN neural network layer as the first feature extraction network layer for drug graph information, with the mathematical expression given by

14 $$\begin{aligned} {H}_{i}=f({H}_{{i}-1},{A})=\sigma \big ({{\widehat{A}}H}_{{i}-1}{W}^{({i}-1)}\big ), \end{aligned}$$

where $H_i$ represents the feature matrix of the molecular graph ${\mathscr {G}}_{\textrm{d}}=({\mathscr {V}}_{\textrm{d}},{\mathscr {E}}_{\textrm{d}})$ for the drug, where $A^{(N \times N)}$ denotes the adjacency matrix. ${{\widehat{A}}}={{\widetilde{D}}}^{-\frac{1}{2}}{{\widetilde{A}}}{{\widetilde{D}}}^{-\frac{1}{2}}.$ represents the symmetric normalization of the adjacency matrix, where ${\widetilde{A}} = A + I_N$, introducing self-loops to the nodes by adding the identity matrix $I_N$, ensuring that node features are included during convolution operations. ${\widetilde{D}} = \sum _j {\widetilde{A}}_{ij}$ is a degree matrix used for normalizing ${\widetilde{A}}$ to prevent the occurrence of gradient explosions. $W^{(i)}$ and $W^{(i-1)}$ represent the weight matrices of the current layer and the previous layer, respectively. $\sigma (\cdot )$ is the ReLU activation function. Subsequently, the graph information extracted from GCN is distilled through multiple residual processes to obtain the refined feature representation of the drug molecule. In mathematical terms, the residual operation is defined as

15 $$\begin{aligned} H_i= & {} F(H_{i-1})=W_i\cdot \sigma \bigl (W^{(i-1)}\cdot f(H_{i-2},A)+b^{(i-1)}\bigr ),\end{aligned}$$

16 $$\begin{aligned} H_i= & {} \sigma (F(H_{i-1})+H_{i-1}), \end{aligned}$$

After that, to reduce the network complexity and improve the training accuracy, we use the graph pooling layer to scale down the redundant information. Finally, after the 2-layer linear layer output of the Mol. encoder, we obtain the feature representation of the drug. The mathematical formulas for the two-step operations are as follows

17 $$\begin{aligned} v_{max}^{\prime }= & {} MaxPool({{\mathscr {V}}}_{d}^{\prime }),\end{aligned}$$

18 $$\begin{aligned} x_d= & {} (W_0v_{max}^{\prime }+b_0)W_1+b_1, \end{aligned}$$

where ${\mathscr {V}}_d'$ represents the node features of the drug graph after the application of GCN. $W_{i \in \{0,1\}}$ and $b_{i \in \{0,1\}}$ denote the weights and biases of the two linear layers, respectively. The obtained vector $x_d \in {\mathbb {R}}^{N'}$ is referred to as the drug molecule node, where $N'$ is the dimensionality of $x_d$.

#### DTG distillation

After encoding through the Mol. encoder, a new drug graph ${\mathscr {G}}_{d}^{\prime }$ is obtained, represented as ${\mathscr {G}}_{d}^{\prime }=(x_{d},{{\mathscr {E}}}_{d}^{\prime })$, and a protein graph ${\mathscr {G}}_{p} = ({\mathscr {V}}_{p}, {\mathscr {E}}_{p})$. The feature fusion of these graphs forms a heterogeneous graph, resulting in an information-rich pool ${\mathscr {G}}_{DTG} = ({\mathscr {V}}_{DTG}, {\mathscr {E}}_{DTG})$, where ${\mathscr {V}}_{DTG} = {\text {concat}}(x_d, {\mathscr {V}}_p)$ and ${\mathscr {E}}_{DTG} = {\text {concat}}({\mathscr {E}}_{d}^{\prime }, {\mathscr {E}}_p)$. The data in these information pools are high-dimensional and redundant. To streamline our data dimensions and expedite model training, the DTG in the information pool will utilize GCN to capture essential feature information. Mathematically, the expression is obtained by

19 $$\begin{aligned} H_{i}^{{\mathscr {G}}_{DTG}}=f\bigl (H_{i-1}^{{\mathscr {G}}_{DTG}}, A_{{\mathscr {G}}_{DTG}}\bigr )=\sigma \bigl ({\hat{A}}_{{\mathscr {G}}_{DTG}} H_{i-1}^{{\mathscr {G}}_{DTG}}W_{{\mathscr {G}}_{DTG}}^{(i-1)}\bigr ), \end{aligned}$$

Then, the DTG is subjected to dimensionality reduction using residual blocks, resulting in the refined drug-protein hetero-network. Mathematically, the expression is as follows

20 $$\begin{aligned} H_{i}^{{\mathscr {G}}_{DTG}}= & {} F(H_{i-1}^{{\mathscr {G}}_{DTG}}) =W_{{\mathscr {G}}_{DTG}}^{(i)}\cdot \sigma \left( W_{i-1}\cdot f \left( H_{i-2}^{{\mathscr {G}}_{DTG}},A_{{\mathscr {G}}_{DTG}}\right) +b_{{\mathscr {G}}_{DTG}}^{(i-1)}\right) ,\end{aligned}$$

21 $$\begin{aligned} H_{i}^{{\mathscr {G}}_{DTG}}= & {} \sigma (F(H_{i-1}^{{\mathscr {G}}_{DTG}}) +H_{i-1}^{{\mathscr {G}}_{DTG}}), \end{aligned}$$

Finally, we separate the refined bipartite graph into drug and protein graphs using a masking approach. Mathematically, this is expressed by

22 $$\begin{aligned} {\mathscr {V}}_{p}^{masked}= & {} Masked({\mathscr {V}}_{DTG}^{\prime }),\end{aligned}$$

23 $$\begin{aligned} {\mathscr {V}}_{d}^{masked}= & {} \sim Masked({\mathscr {V}}_{DTG}^{\prime }), \end{aligned}$$

where ${\mathscr {V}}_d^{{\text {masked}}}$ and ${\mathscr {V}}_p^{{\text {masked}}}$ represent the separated sets of drug nodes and protein nodes, respectively.

#### DTA score

At the final stage of the model, the separated bipartite graphs flow into their respective data channels, resulting in the drug representation $X_d^{(final+1)}$ and the protein representation $X_p^{(final+1)}$. The mathematical expressions are given by

24 $$\begin{aligned} X_{d}^{final}= & {} concat(X_{j+1},{\mathscr {G}}({\mathscr {V}}_{d}^{masked}, {\mathscr {E}}_{d}^{masked})),\end{aligned}$$

25 $$\begin{aligned} X_d^{final+1}= & {} MaxPool(X_d^{final}),\end{aligned}$$

26 $$\begin{aligned} X_{p}^{final}= & {} {\mathscr {G}}\bigl ({\mathscr {V}}_{p}^{masked}, {\mathscr {E}}_{p}^{masked}\bigr ),\end{aligned}$$

27 $$\begin{aligned} X_p^{final+1}= & {} Linear\Big (ReLU\Big (Pooling(X_p^{final})\Big )\Big ), \end{aligned}$$

To improve predictive accuracy, we combine the drug features before feature fusion with those obtained after the separation of the bipartite graph. This integration results in a new set of drug features. Subsequently, we employ a fully connected block to concatenate these drug features with protein features for the prediction of protein-drug affinity values. Mathematically, the expression is formulated as

28 $$\begin{aligned} DTAscore=Linear\Big (concat\big (X_d^{final+1},X_p^{final+1}\big )\Big ), \end{aligned}$$

## Results and discussion

### Evaluation indicators

Many metrics exist for assessing model performance and capacity in current research in the DTA/DTI field. However, the selection of different metrics for different research questions with different contextual information often leads to different measures. Therefore, we use mean squared error (MSE), root mean square error (RMSE), Pearson, Spearman, consistency index (CI)^51^ and $r^2$ (coefficient of determination) to assess the performance of our models.

**MSE**: MSE is used to measure the squared average difference between the model’s predicted values and the actual observed values. For a set of actual observed values (or target values) $y_i$ and their corresponding predicted values (or model outputs) $\widehat{y_i}$, the calculation of MSE is as follows:

29 $$\begin{aligned} MSE=\frac{1}{n}\sum _{i=1}^n(y_i-\widehat{y_i})^2, \end{aligned}$$

**RMSE**: A measure of the square root of the mean squared difference between the predicted and actual values.

30 $$\begin{aligned} RMSE=\sqrt{\frac{1}{n}\sum _{i=1}^n(y_i-\widehat{y_i})^2}, \end{aligned}$$

**Pearson**: Measures the linear correlation between the predicted value X and the underlying true value Y.

31 $$\begin{aligned} Pearson=\frac{E(XY)-E(X)E(Y)}{\sqrt{E(X^2)-E^2(X)}\sqrt{E(Y^2)+E^2(Y)}}, \end{aligned}$$

where, cov(X,Y) is the covariance between the predicted value and the underlying fact, $\sigma (X)$ is the standard deviation of X, and $\sigma (Y)$ is the standard deviation of Y. $\mu _X$, $\mu _Y$ are the mean values of the distributions of X,Y, respectively.

**Spearman**: A statistic obtained by arranging the sample values of two random variables in order of their data magnitude, using the ranks of the individual sample values instead of the actual data.

32 $$\begin{aligned} \rho =\frac{\frac{1}{n}\sum _{i=1}^{n} A \cdot B }{\sqrt{\left( \frac{1}{n} \sum _{i=1}^{n} A^2\right) \cdot \left( \frac{1}{n}\sum _{i=1}^{n}B^2\right) }}, \end{aligned}$$

where $R({\hat{y}}_{i})$ is the predicted value ranking, $R(y_i)$ is the true value ranking, $\overline{{{R({\hat{y}})}}}$ is the average of the predicted value ranking, and $\overline{{{R(y)}}}$ is the average of the true value ranking, $A = R(y_{i})-\overline{R(y)}$, $B = R(\hat{y_{i}})-\overline{R({\hat{y}})}$.

**CI**: Measures the probability of correctly predicting unequal pairs according to the order.

33 $$\begin{aligned} CI=\frac{1}{Z}\sum _{\delta _i>\delta _j}h\bigl (x_i-x_j\bigr ), \end{aligned}$$

where $x_i$ is the predicted value of the larger affinity $\delta _i$, $x_j$ is the predicted value of the smaller affinity $\delta _j$, Z is the number of unequal pairs as the normalization constant, and h(x) is the step function^33^:

34 $$\begin{aligned} h(x)=\left\{ \begin{array}{ll} 1, &{} \quad {\text {if }}x>0\\ 0.5, &{} \quad {\text {if }}x=0\\ 0, &{} \quad {\text {if }}x<0 \end{array}\right. , \end{aligned}$$

This metric measures whether the predicted binding affinity values for any drug-target pair are predicted in the same order as their true values. We used paired t-tests to perform statistical significance tests with 95% confidence intervals.

${\varvec{r}}^{{\varvec{2}}}$: Given the varying scales of different datasets, it’s challenging to compare them using metrics like MSE and RMSE mentioned above. This metric calculates the $\hbox {R}^{2}$ value with a reference to the mean model for comparing the quality of models. The formula for calculating the $r^2$ is as follows:

35 $$\begin{aligned} r^2=1-\frac{\sum _i\left( {\hat{y}}_{i}-y_{i}\right) ^2}{\sum _i \left( {\overline{y}}-y_{i}\right) ^2}, \end{aligned}$$

where ${\hat{y}}_{i}$ is the predicted value, $y_{i}$ the real value, and ${\overline{y}}$ the mean of the real values.

### Experiment setup

We evaluate the performance of our proposed model on benchmark datasets^31,32^. We will use the same nested cross-validation as the DeepDTA^21^ method to determine the best parameters for the validation and test sets. To train the generalized linear model with enhanced generalization, we randomly partition the dataset into 6 equal parts (4:1:1), designating one part as the independent test set. The remaining parts are utilized for hyperparameter tuning through 5-fold cross-validation. We conducted special processing for the KIBA dataset. To accelerate model training, we divided the KIBA dataset into four parts and trained each of the four subsets with identical parameters. KronRLS^33^, Simboost^14^, and others use folds with the same settings as the training, test, and validation sets for a fair comparison.

We set different filter sizes for drug compounds and proteins instead of generic sizes for the experiments because they have different contextual representations. In Table 2, the hyperparameter combination corresponding to the best CI score provided on the validation set is selected as the best hyperparameter combination for modeling the test set.

**Table 2:** Hyperparameter settings for GEFormerDTA.

Parameters	Value
Number of res. blocks	[2; 3; 4]
Number of GCNConv. Blocks	[1; 2]
NUM_EPOCHS	2000
Hidden Neurons	[256; 512]
TRAIN_BATCH_SIZE	[64; 128]
TEST_BATCH_SIZE	256
DROPOUT	[0.2; 0.5]
OPTIMIZER	Adam
LR	[0.0005; 0.001; 0.01]

**Table 3:** Predicted binding affinity for the Davis independent test set (“underlined” means suboptimal; “bolded” means optimal).

Method	$\downarrow$MSE	$\downarrow$RMSE	$\uparrow$Pearson	$\uparrow$Spearman	$\uparrow$CI	$\uparrow r^2$
KronRLS^∗^^25^	0.443	0.665	–	0.624	0.847	0.473
SimBoost^∗^^25^	0.277	0.526	–	0.694	0.891	0.670
DeepDTA^21^	**0.196**	**0.442**	0.850	**0.845**	0.866	0.712
DeepCDA^∗^^22^	0.248	–	0.857	–	$\underline{0.891\pm 0.003}$	$0.649\pm 0.009$
GraphDTA-GCNNet^51^	0.293	0.541	0.797	0.660	0.863	0.635
GraphDTA-GINNet^51^	0.261	0.511	0.821	0.691	0.884	0.674
GLFA^52^	0.241	0.491	0.839	0.693	0.886	0.699
GEFA^52^	0.250	0.500	0.832	0.69460	0.887	0.688
GEFormerDTA	0.212	0.461	**0.889**	0.69465	**0.895**	**0.735**

* Reference original data.

### Results

#### Comparison experiments

In Tables 3 and 4, KronRLS, SimBoost, DeepDTA, and DeepCDA are mainly based on token-based SMILES representations and token-based FASTA sequence representations, while GraphDTA-GCNNet, GraphDTA-GINNet, GLFA, and GEFA are mainly base on representations of drug graphs or protein graphs.

In Table 3, We report some work on Transformer graph early fusion methods on the benchmark datasets Davis and KIBA. Our proposed method achieves the best performance among all listed methods, which is in line with our expectations. To validate the validity and feasibility of the GEFormerDTA method, we evaluated and compared the predictive accuracy of different state-of-the-art binding affinity regression models. The performance of the GEFormerDTA model compared with existing baseline models on the Davis independent test set is depicted in Table 3. The proposed method achieved good results in three of the six metrics. The change in the CI metric is less significant compared to the best-performing existing methods, showing an improvement of only 0.4 percentage points. The Pearson correlation coefficient and $r^2$ value increased by 3.2 and 2.3% points, respectively. Our ESC drug encoder fully uses information such as atomicity center encoding, chemical bond encoding, and spatial information encoding in the drug feature map. MSE, RMSE, and Pearson did not yield satisfactory results, being 1.7, 1.8, and 15 percentage points lower than the optimal performance across all baselines, respectively. Transformer has global information awareness, which is very beneficial to obtain complete drug features containing richer informa- tion than GCN. This also demonstrates the advantage of applying a Transformer to graph problems.

Table 4 compares the performance of the GEFormerDTA model with the existing baseline model using the KIBA independent test set. We conducted experiments with our model on four subsets of the KIBA dataset. The proposed method showed good performance in the split3 subset, achieving strong results across four metrics (MSE = 0.06, RMSE = 0.244, Spearman = 0.884, $r^2$ = 0.898). The CI metric performed best in the split2 subset with a value of 0.896. Our model, GraphDTA-GINNet, achieved the best result in the Pearson metric, with a score of 0.872. Compared to the highest levels of existing methods, the change in the Pearson metric is minimal, with an improvement of only 0.16% points in the split1 subset. The maximum improvement in the $r^2$ metric, when compared to other models, is 4.5% points. In Table 4, GEFormerDTA outperforms baseline models in terms of performance, and the comparison with GEFA in Table 3 highlights the reliability and effectiveness of drug encoding in our method. In recent articles, CI has been used as the primary evaluation metric in models. Although we did not achieve the best performance in some metrics, our model achieved the best CI on two datasets.

**Table 4:** Predicted binding affinity for the KIBA independent test set (“underlined” means suboptimal; “bolded” means optimal).

Method		$\downarrow$MSE	$\downarrow$RMSE	$\uparrow$Pearson	$\uparrow$Spearman	$\uparrow$CI	$\uparrow$ $r^2$
KronRLS^∗^^22^		0.411	–	–	–	$0.782\pm 0.0009$	$0.342\pm 0.001$
SimBoost^∗^^22^		0.222	–	–	–	$0.836\pm 0.001$	$0.629\pm 0.007$
DeepDTA^21^		0.082	0.287	0.710	0.645	0.849	0.504
DeepCDA^∗^^22^		0.176	–	0.855	–	$\underline{0.889\pm 0.002}$	$0.682\pm 0.008$
GraphDTA-GCNNet^51^		0.188	0.433	0.856	0.845	0.862	0.724
GraphDTA-GINNet^51^		0.163	0.404	**0.872**	0.863	0.873	0.760
GLFA^52^	split_avg	0.215	0.463	0.822	0.826	0.858	0.673
	split 1	0.227	0.476	0.829	0.818	0.852	0.685
	split 2	0.226	0.476	0.850	0.842	0.867	0.719
	split 3	0.187	0.432	0.827	0.835	0.862	0.679
	split 4	0.221	0.470	0.782	0.808	0.851	0.609
GEFA^52^	split_avg	0.217	0.466	0.821	0.820	0.855	0.669
	split 1	0.236	0.486	0.822	0.809	0.849	0.671
	split 2	0.220	0.469	0.852	0.840	0.864	0.725
	split 3	0.191	0.438	0.826	0.831	0.862	0.673
	split 4	0.222	0.471	0.783	0.800	0.844	0.607
GEFormerDTA	split_avg	0.081	0.284	0.835	0.871	0.877	0.844
	split 1	0.091	0.302	0.821	0.8819	0.870	0.805
	split 2	0.099	0.314	0.832	0.8817	**0.896**	0.819
	split 3	**0.060**	**0.244**	0.851	**0.884**	0.879	**0.898**
	split 4	0.076	0.276	0.837	0.839	0.864	0.854

* Reference original data.

**Figure 5:**
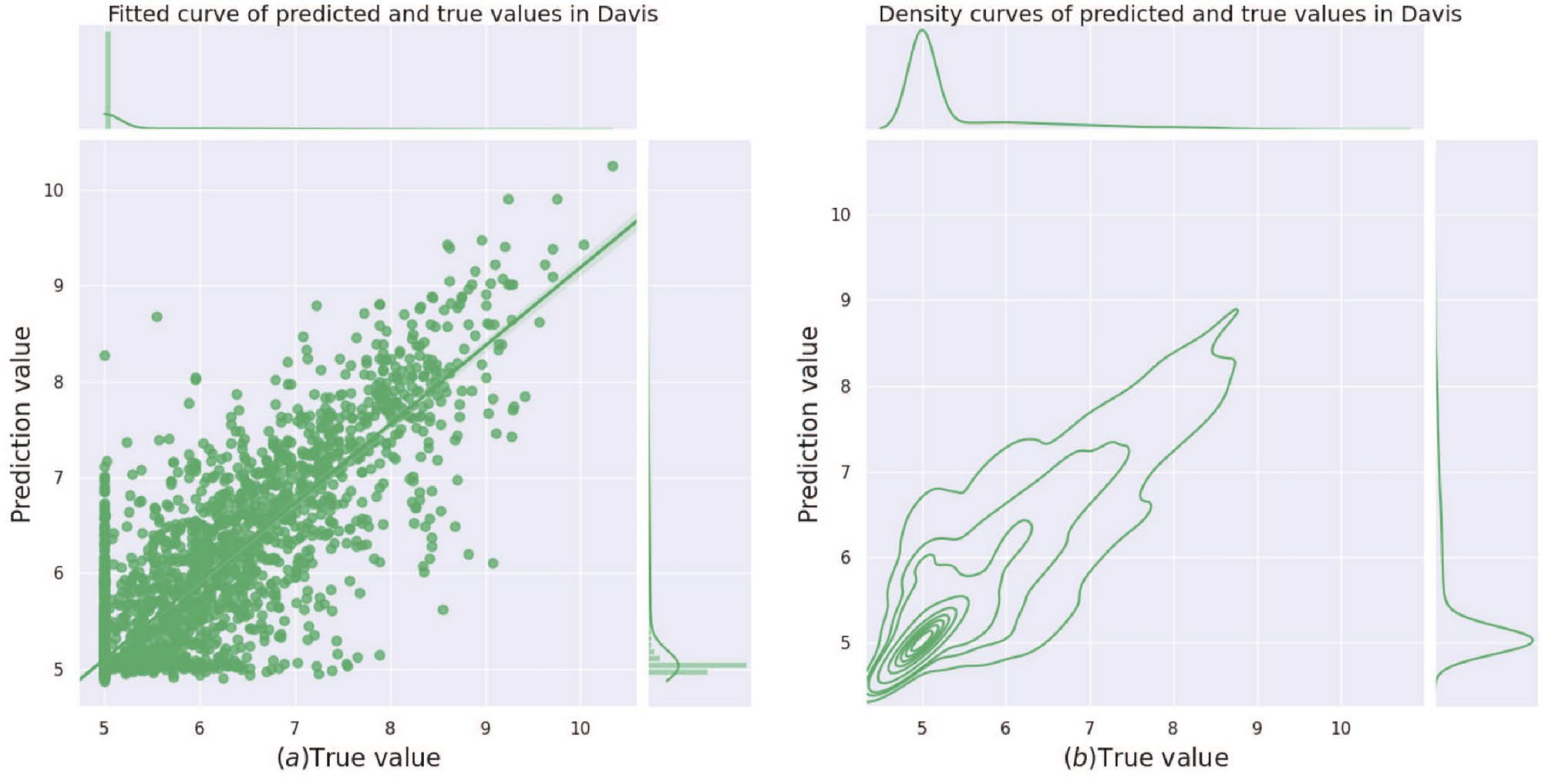
(**a**) Linear regression fitted straight lines for true and predicted values on the Davis dataset. (**b**) Kernel density estimation plots of the true and predicted values on the Davis data set. where the horizontal coordinates indicate the true binding affinity, and the vertical coordinates indicate the predicted binding affinity. The upper and right bars show the distribution characteristics of the sample size.

To visually represent the predictive performance of our model, Fig. 5a illustrates the fit of the predicted binding affinity values to the true values on the Davis dataset. The scatter plot shows that data points are distributed on both sides of the line ${\hat{y}}=y$, indicating a reasonable fit. Figure 5b displays the kernel density estimates of the predicted binding affinity values compared to the true values. The dense distribution of curves suggests a high degree of data density. The circular curves generally have an oval shape, and their long axes roughly align with the curve ${\hat{y}}=y$.

**Figure 6:**
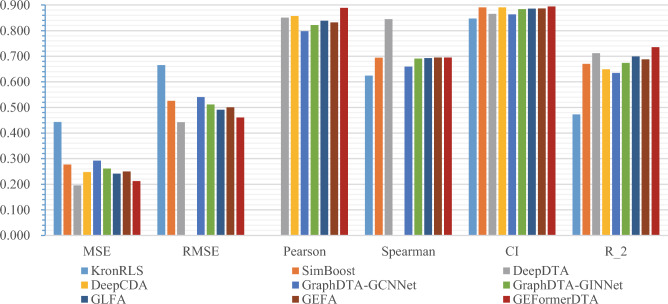
Comparison of the levels of our method and other methods on the Davis dataset under the six-evaluation metrics.

**Figure 7:**
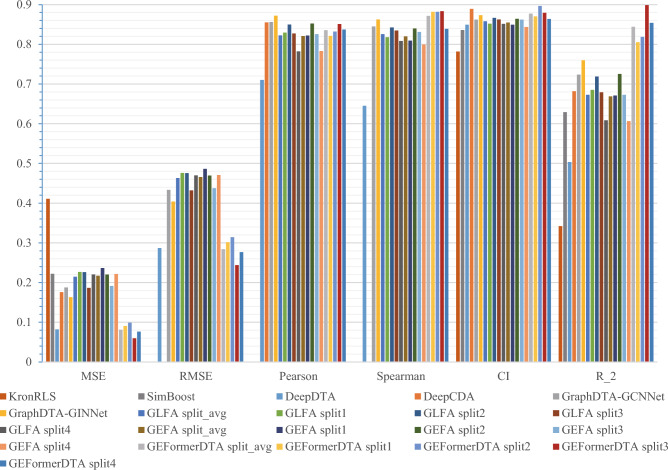
Comparison of the levels of our method and other methods on the KIBA dataset under the six-evaluation metrics.

Figures 6 and 7 show the performance comparison of our method with other methods on two gold standard datasets. As can be seen from the figure, the CI metric improves on both datasets compared to the baseline model. Among the six evaluation metrics, the proposed method significantly improves Pearson on four subsets of KIBA. In contrast, on the Davis data set, the improvement of $r^2$ is more obvious, which shows that our model has stronger generalization ability on the Davis data set.

#### Ablation studies

**Figure 8:**
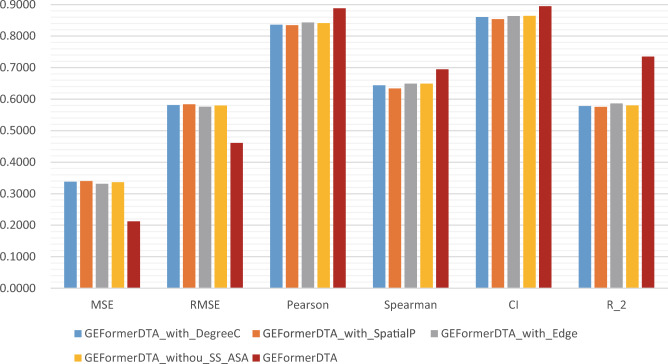
Comparison of the levels of our method and other methods on the Davis dataset under the six-evaluation metrics.

It is well known that the way drug data are encoded is important for the predictive performance of the model during the study of DTA. To verify the importance of each substructure of drug coding in the drug preprocessing stage and the effect on the model performance, we performed ablation experiments on each substructure. In Table 5, the GEFormerDTA model without encoding substructures (first three rows) all performed worse than the model with both three encoding substructures. The GEFormerDTA model without protein secondary structure and accessible surface area feature encoding (fourth row) perform worse than the model with protein structural features. This is enough to show that the protein structure has a positive effect on improving the performance of the proposed model. In order to visually represent the progress of centroid encoding, edge encoding, and spatial encoding more intuitively, we present the results from Table 5 in the form of bar charts in Fig. 8.

**Table 5:** Ablation experiments based on drug coding modalities in the Davis independent prediction dataset (“underlined” means suboptimal; “bolded” means optimal).

Method	$\downarrow$MSE	$\downarrow$RMSE	$\uparrow$Pearson	$\uparrow$Spearman	$\uparrow$CI	$\uparrow r^2$
GEFormerDTA_with_DegreeC	0.3383	0.5816	0.8366	0.6436	0.8605	0.5779
GEFormerDTA_with_SpatialP	0.3404	0.5834	0.8346	0.6341	0.8539	0.5753
GEFormerDTA_with_Edge	0.3315	0.5758	0.8436	0.6494	0.8636	0.5864
GEFormerDTA_withou_SS_ASA	0.3364	0.5800	0.8411	0.6493	0.8641	0.5803
GEFormerDTA	**0.2124**	**0.4609**	**0.8885**	**0.6946**	**0.8947**	**0.7350**

## Conclusion

In this paper, we propose a novel deep learning approach using Transformer to solve graph structure data to solve the problem of drug affinity prediction, which can accelerate the development of physical drugs and repurposing of old drugs. After our analysis of model prediction results, we found that GEFormerDTA is very effective in grasping drug molecule graph structure information (degree centrality encoding, atomic space encoding and edge encoding) without prior knowledge for model performance improvement.

Considering the potential representation changes due to protein metastability during the binding process, we use an early fusion approach between drug and target. The early fusion technique transforms the parallel processing of drug and protein into a serial processing of affinity problems by integrating drug representation into protein representation co-learning. The interpretability of the model can be enhanced by using the self-attentive values of the hidden features of protein nodes as edge weights connecting drug nodes and residue nodes in the target protein graph, which quantifies which residues play a role for the binding process and the level of contribution of each residue. Early fusion is shown to be more competitive than late fusion by comparative tests. Exploiting the molecular map structural information of a drug is more advantageous than solo thermal coding. Experiments show that our method outperforms other advanced methods.

However, there are still many potential limitations to our current work. Our approach has not been able to address the conformational changes caused by protein-drug contact. The study of protein conformational changes is an important area of current biological research, which provides the basis for in-depth exploration in the life sciences. In addition, the study of protein conformational change mechanisms may also have important implications for drug development, disease prevention and control, and health management. Therefore, there is still great potential and space for future research in protein conformational changes. If we can learn residue-edge attachment changes, we can explain the conformational changes arising from drug-protein binding. Our approach is portable and scalable. In the prediction of protein-RNA interactions, we can share the structural coding information of some of the proteins in our work and additionally can incorporate the electrostatic patch (EP) information of the proteins.

## Data Availability

The datasets generated and/or analysed during the current study are available in the github repository, https://github.com/CellNest/GEFormerDTA/.

## Abbreviations

SS: Secondary structure
ASA: Accessible surface area
DTI: Drug-target interactions
DTA: Drug-target affinity
GCN: Graph convolutional neural
ESC: Encoder for feature extraction for edge coding, spatial position coding and centrality coding
